# Topology-Dependent Compression and Energy Absorption of 3D-Printed Resin Scaffolds Filled with Polyurethane Foam

**DOI:** 10.3390/polym18131584

**Published:** 2026-06-25

**Authors:** Yi Jie, Yongcheng Hong, Yajiu Zhang

**Affiliations:** School of Civil Engineering and Transportation, Guangzhou University, Guangzhou 510006, China; jieyi2023@e.gzhu.edu.cn (Y.J.); 13037271132@163.com (Y.H.)

**Keywords:** interpenetrating phase composites, triply periodic minimal surface structures, additive manufacturing, energy absorption, polyurethane foam

## Abstract

Lightweight resin lattice structures are prone to instability and failure under compressive loading, which leads to limited load bearing capacity and energy absorption performance. In this study, tough resin triply periodic minimal surface (TPMS) lattice scaffolds were fabricated using stereolithography-based 3D printing, and polyurethane foam (PUF) was subsequently infiltrated into three representative topologies, namely Schwarz Primitive (P), I-Wrapped Package (IWP), and Gyroid (G), to form interpenetrating phase composites (IPC). Quasi-static compression results show that PUF infiltration significantly improves the compressive response of all IPC architectures. The stress level in the plateau region is increased, while the magnitude of local stress drops is reduced, leading to a more stable progressive compression behavior. By comparing the stress–strain responses of IPC with the linear superposition of the pure resin scaffold and PUF phases, it is found that the actual energy absorption of IPC exceeds the predicted additive response, indicating a pronounced synergistic effect between the two phases. Among them, the IWP-based IPC achieves a specific energy absorption of 11.72 J/g. These results demonstrate that interpenetrating phase architectures can maintain lightweight characteristics while enhancing load bearing stability and energy absorption efficiency, providing useful guidance for topology selection and lightweight design of TPMS-based energy absorbing composite structures.

## 1. Introduction

Lightweight design and high energy absorption are two critical requirements for structural materials in aerospace, rail transportation, and automotive protection applications [1,2]. Conventional monolithic resin materials typically exhibit high density and low specific performance, and their inherent brittleness often leads to sudden fracture under impact loading, making it difficult to simultaneously achieve both lightweight characteristics and high energy absorption capacity [3,4].

Lattice structures achieve a significant reduction in density by periodically arranging solid materials, while maintaining high specific strength and stiffness [5,6]. Additive manufacturing, particularly stereolithography, enables the high-precision fabrication of complex resin lattice architectures [7,8]. However, even when relatively ductile photocurable resins are employed, low-density lattice structures fabricated from them still face considerable challenges [9,10]. At low volume fractions, these structures are prone to buckling or shear instability, leading to a sharp stress drop after peak load, with the plateau region either absent or severely shortened, which in turn significantly limits their energy absorption capacity [11,12]. Therefore, overcoming catastrophic failure in monolithic lattice structures, extending the stable plateau region, and improving energy absorption efficiency remain critical issues that urgently need to be addressed.

Interpenetrating phase composites (IPC) provide a promising strategy for addressing these challenges. IPC are formed by infiltrating a matrix phase into a three-dimensional interconnected porous scaffold, resulting in a co-continuous structure in which both phases remain spatially continuous [13,14]. The matrix phase provides lateral confinement to the scaffold, suppressing local buckling, while the interfacial boundaries act as load transfer pathways that redistribute local stresses throughout the structure, thereby delaying crack initiation and propagation [15,16]. As a result, IPC designs can significantly enhance the strength and energy absorption capacity of monolithic lattice structures [17,18].

In recent years, the IPC strategy has been validated in a variety of lattice systems. Wang et al. [19] infiltrated epoxy resin into stainless steel lattices, achieving increases in yield strength of 363–1109% and improvements in specific energy absorption of 27–290%. Even for brittle ceramics, the incorporation of an epoxy matrix to form IPC can enhance the peak strength by more than 30 times [20]. When combined with triply periodic minimal surface (TPMS) architectures, the advantages of IPC are further amplified, with ceramic epoxy TPMS IPC exhibiting an increase in specific energy absorption of up to 1109% [21]. These studies demonstrate that the IPC concept provides an effective approach for overcoming the performance limitations of conventional monolithic lattice structures.

However, the current understanding remains insufficient in two main aspects. First, most existing studies focus on a single topology or a relatively narrow range of volume fractions, lacking systematic comparisons among multiple representative architectures over a wide range of relative densities [22,23]. Since different topologies exhibit fundamentally distinct deformation mechanisms and stress transfer pathways, which directly govern the reinforcement efficiency of IPC [24,25], the absence of a comprehensive investigation makes it difficult to establish reliable guidelines for structural optimization and performance maximization. Second, polyurethane foam (PUF) is well known for its excellent deformability and compatibility with various material systems [26,27]. Using PUF as a matrix phase to infiltrate resin scaffolds is expected to significantly enhance toughness while maintaining lightweight characteristics. However, its role as a matrix phase in reinforcing resin-based composite systems has not been sufficiently explored, and its influence on specific strength, specific energy absorption, and toughness remains unclear. This gap in knowledge provides the direct motivation for the present study.

Based on the above considerations, different TPMS topologies exhibit distinct geometric characteristics and load transfer mechanisms, topology-dependent mechanical behavior is expected even at identical relative densities. In this work, polymer interpenetrating phase composites were fabricated by using additively manufactured photopolymer TPMS lattices as the reinforcing phase and polyurethane foam (PUF) as the matrix phase. Through quasi-static compression tests, the compressive response and failure mechanisms of both monolithic resin scaffolds and their corresponding IPC were systematically investigated across three representative TPMS topologies Schwarz Primitive (P), I-Wrapped Package (IWP), and Gyroid (G) and three relative densities (20%, 25%, and 30%).

The objectives of this study are to (i) elucidate the effects of topology and relative density on the compressive behavior and failure modes of monolithic resin scaffolds; (ii) reveal the strengthening mechanism of the PUF matrix within IPC; and (iii) quantitatively evaluate the enhancement effect of PUF infiltration by comparing IPC with their corresponding resin scaffolds, thereby clarifying its role in improving specific strength, specific energy absorption, and lightweight performance.

TPMS architectures were selected because their smooth and continuous surfaces reduce stress concentration, promote uniform load transfer, and facilitate complete infiltration of the polyurethane foam phase. These characteristics make TPMS structures particularly suitable for the design of interpenetrating phase composites with enhanced energy absorption performance.

## 2. Materials and Methods

### 2.1. Structure Design

The reinforcement phase of the IPC adopts three types of lattice structures designed based on triply periodic minimal surfaces (TPMS), which are mathematically defined surfaces that repeat periodically in three orthogonal spatial directions (x, y, and z) while maintaining zero mean curvature.

**Figure 1:**
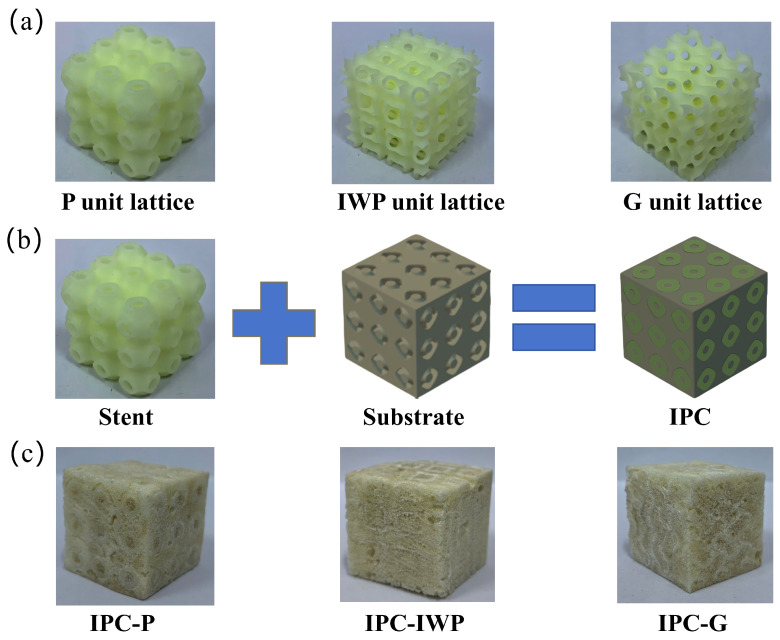
(**a**) Photos of 3D-printed tough resin lattice scaffolds with P, IWP, and G structures; (**b**) IPC design; (**c**) photos of IPC with three architectures.

In the design of TPMS porous scaffolds, the P and IWP architectures were deliberately selected in Figure 1. The inherent symmetry of the P and IWP topologies promotes a more uniform distribution of forces, resulting in a more homogeneous stress field throughout the scaffold [28,29]. Such uniformity is crucial for maintaining structural integrity, as it helps alleviate stress concentrations that may otherwise lead to deformation or catastrophic failure. The G structure features a continuously smooth surface, which is beneficial for the infiltration of the secondary phase. Therefore, this design strategy aims to ensure sufficient mechanical strength of the scaffold while enabling uniform filling during polyurethane casting.

The reinforcement phase in TPMS is defined mathematically using a level set approximation method, which allows the extraction of an isosurface at a specified iso value. The related mathematical expressions are provided in Equations (1)–(3) [7,23].

(1) P(x,y,z)=cos(x)+cos(y)+cos(z)=C

(2) $$\begin{array}{l} IWP(x,y,z)=2\left[\cos(x)\cos(y)+\cos(y)\cos(z)+\cos(z)\cos(x)\right]\\ -\left[\cos(2x)+\cos(2y)+\cos(2z)\right]=C \end{array}$$

(3) G(x,y,z)=sin(x)cos(x)+sin(z)cos(y)+sin(y)cos(z)=C

In these equations, *P*(*x*, *y*, *z*), *IWP*(*x*, *y*, *z*), and *G*(*x*, *y*, *z*) denote the implicit functions of the *P*, *IWP*, and *G* structures along the spatial coordinates, respectively. When *C* = 0, the resulting surface corresponds to a minimal surface with zero mean curvature. By varying the parameter *C*, the geometric configuration can be adjusted, which, in turn, influences the resulting volume fraction of the structure. This parametric approach enables the design of porous scaffolds with tunable mechanical properties.

The volume fraction reported in this study refers to the solid volume fraction of the printed resin scaffold within the total specimen volume, which is equivalent to the relative density of the lattice structure.

### 2.2. Design and Fabrication of IPC Specimens

The reinforcement phase of the IPC adopts three types of lattice structures designed based on triply periodic minimal surfaces (TPMS), namely the P, IWP, and G configurations.

All architectures in this study were constructed from unit cells with a side length of 10 mm, arranged in a 3 × 3 × 3 periodic configuration to form cubic specimens with overall dimensions of 30 × 30 × 30 mm^3^. All geometric models were generated using Rhino 7 software. The resin scaffolds were fabricated using a tough photosensitive resin (Goda 8228U, Dongguan, China) via a stereolithography printer from CREALITY (Shenzhen, China). Goda 8228U is a commercial tough photocurable resin mainly composed of multifunctional acrylate oligomers, reactive acrylate monomers, photoinitiators and proprietary additives. A cross-linked polyurethane acrylate polymer network was formed through photopolymerization. The processing parameters were set as follows: XY resolution of 0.05 mm, scanning time of 1–4 s per layer, and a laser wavelength of 355 nm. After printing, the samples were cleaned by ultrasonic washing in absolute ethanol.

**Figure 2:**
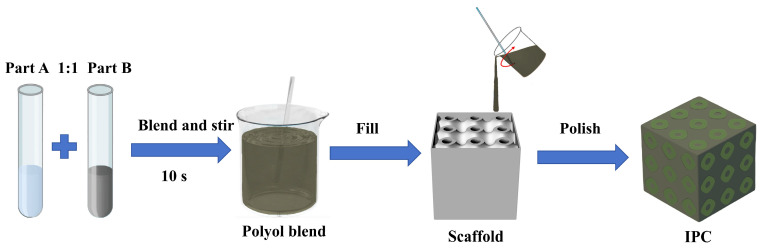
Fabrication process of interpenetrating phase composite specimens.

The fabrication process of the interpenetrating phase composite (IPC) is illustrated in Figure 2. The composite foam was prepared using a two component polyurethane system (FM 1380) via a mixing and foaming process. Component A consists of a polyether polyol (white component), while Component B is a polyisocyanate (black component). After mixing components A and B, a foaming reaction occurred immediately. The generated polyurethane foam expanded and infiltrated the interconnected pores of the resin scaffold before curing, resulting in a fully interpenetrating phase architecture. The cured foam has a density of 150 kg/m^3^. Component A and Component B were first weighed at a mass ratio of 1:1. The two components were then mixed thoroughly and stirred vigorously for 10 s before being quickly poured into a silicone mold. During the process, a fine syringe needle was used to remove trapped air bubbles to minimize void defects.

The tough resin scaffolds were placed inside a silicone mold with dimensions of 35 × 35 × 35 mm^3^. The prepared foam mixture was then poured into the mold to fully infiltrate and fill all voids within the lattice structure. After curing, the IPC specimens were trimmed and cut to obtain the final test samples.

### 2.3. Morphological Characterization

The fracture morphologies of the TPMS-based interpenetrating phase composites (IPCs) were characterized using scanning electron microscopy (SEM, SU8010, Hitachi, Tokyo, Japan). Prior to SEM examination, all samples were sputter-coated with a thin layer of gold to improve surface conductivity and reduce charging effects. SEM images were acquired at different magnifications to investigate the filling state of the polyurethane foam (PUF), the interfacial bonding between the resin scaffold and the foam phase, and the fracture characteristics of different TPMS topologies (P, IWP, and G).

### 2.4. Density Measurement

The densities of the printed resin scaffolds and the corresponding interpenetrating phase composites (IPC) were determined using the mass-to-volume method. For each topology and volume fraction, three specimens were measured and the average value was reported. The specimen dimensions were measured using a digital caliper, and the specimen volume was calculated from the measured dimensions. The bulk density was calculated according to Equation (4):

(4) $$\rho =\frac{M}{V}$$

where ρ is the density of the specimen, M is the measured mass, and V is the specimen volume.

For the printed resin scaffolds, the relative density was determined using Equation (5):

(5) $$\bar{\rho }=\frac{\rho^{*}}{\rho^{s}}$$

where $\bar{\rho }$ is the relative density, $\rho^{*}$ is the measured density of the scaffold, and $\rho^{s}$ is the density of the solid photosensitive resin; $\rho^{s}$ was provided by the manufacturer’s technical data sheet.

The relative density of the TPMS scaffold was calculated using the density of the fully solid photocurable resin ($\rho^{s}$ = 1.15 g/cm^3^).

The measured relative densities were in good agreement with the designed values of 20%, 25%, and 30%, indicating satisfactory fabrication accuracy. After polyurethane foam infiltration, the specimen density increased by approximately 45–55% while maintaining lightweight characteristics due to the low density of the foam phase.

The measured densities and relative densities of the resin scaffolds, together with the densities of the corresponding IPC specimens, are summarized in Table 1.

### 2.5. Quasi-Static Compression Tests

Cubic specimens with dimensions of 30 mm × 30 mm × 30 mm were used for compression testing. Uniaxial compression tests were conducted on monolithic scaffolds and composite specimens using a WANCE universal testing machine (50 kN, TSE305D, Shenzhen, China) under displacement control mode. Compression tests were conducted according to ASTM D695-15. The crosshead was maintained at a constant speed of 1.8 mm/min, corresponding to a nominal strain rate of 0.001 s^−1^. The tests were terminated at a nominal compressive strain of 0.7, at which point all specimens had entered the densification stage. The stress–strain responses were recorded, and the peak stress was extracted for analysis. To comprehensively evaluate the mechanical performance, energy absorption (EA) was adopted as a key metric, defined as follows in Equation (6):

(6) $$EA=\int_{0}^{d}F(x)dx$$

where F(x) denotes the compressive force and d represents the effective displacement at the end of the plateau region. To account for variations in specimen mass, specific energy absorption (SEA) was additionally introduced, defined as follows in Equation (7):

(7) $$SEA=\frac{EA}{M}=\frac{\int_{0}^{d}F(x)dx}{M}$$

where M denotes the mass of the specimen.

## 3. Results and Discussion

### 3.1. Microstructural Characterization

**Figure 3:**
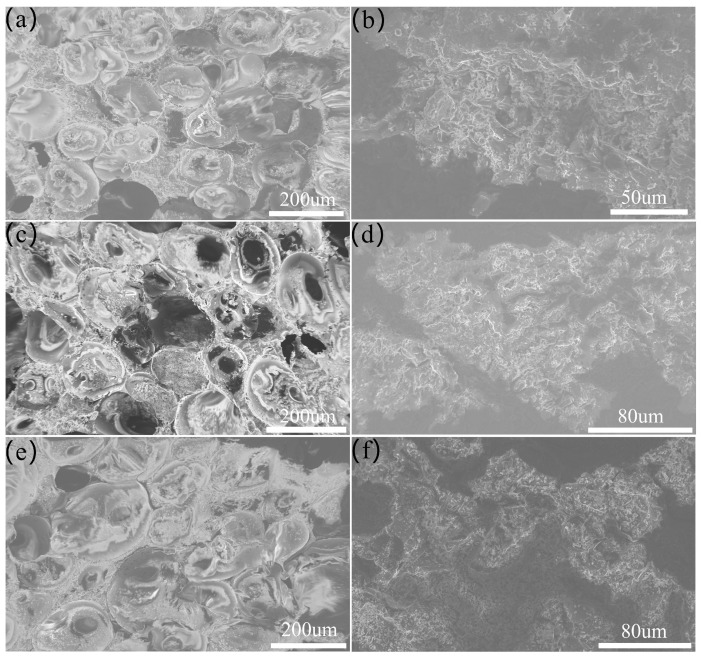
SEM micrographs of the fracture surfaces of a volume fraction of 20% TPMS-based interpenetrating-phase composites (IPC): (**a**,**b**) IPC-P20; (**c**,**d**) IPC-IWP20; (**e**,**f**) IPC-G20.

Figure 3 presents SEM images of the fracture surfaces of TPMS-based interpenetrating phase composites (IPC) with a volume fraction of 20%. Low-magnification images (Figure 3a,c,e) show that the polyurethane foam uniformly filled the interconnected pores of the resin TPMS scaffolds, forming a continuous interpenetrating phase architecture without obvious macroscopic voids or unfilled regions. This demonstrates that the foaming and infiltration process was effective for all three TPMS topologies.

Figure 3b,d,f further reveal intimate contact between the polyurethane foam and the resin framework. No obvious interfacial gaps or large-scale debonding were observed, indicating good interfacial compatibility between the two phases. The continuous foam phase provides lateral support to the resin skeleton during compression, while the interconnected resin framework effectively constrains the deformation of the foam. Such a co-continuous architecture is beneficial for efficient stress transfer and contributes to the synergistic enhancement in compressive strength and energy absorption observed in the IPC specimens.

The SEM observations provide direct microstructural evidence for the synergistic strengthening mechanism of the IPC. During compression, the resin TPMS scaffold serves as the primary load-bearing framework, whereas the polyurethane foam delays local buckling and stabilizes the deformation of the scaffold through lateral confinement. The intimate contact between the two continuous phases facilitates stress transfer across the interface, resulting in higher compressive strength and energy absorption than those predicted by the linear superposition of the individual constituents.

### 3.2. Compressive Mechanical Properties of P, IWP, and G Scaffold Metamaterials

**Figure 4:**
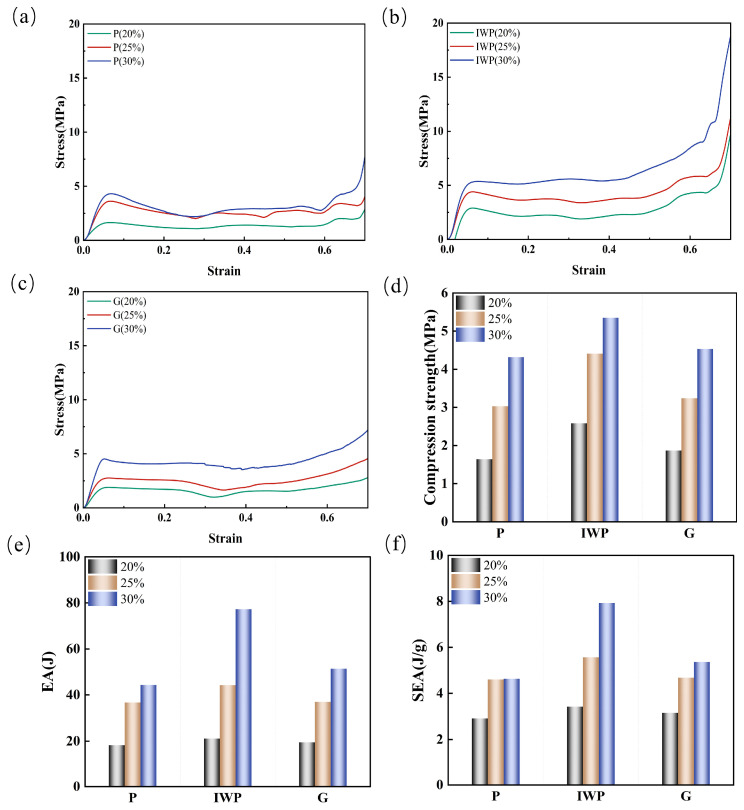
(**a**–**c**) Quasi-static compressive stress–strain curves of tough resin metamaterials with P, IWP, and G topologies at volume fractions of 20%, 25%, and 30%. (**d**) Comparison of peak strength. (**e**,**f**) Comparison of energy absorption (EA) and specific energy absorption (SEA).

Figure 4a–c presents the quasi-static compressive stress–strain responses of the monolithic tough resin metamaterials. The corresponding peak strength and volumetric energy absorption capacity are summarized in Figure 4d and Figure 4e,f, respectively.

For the P structure with volume fractions of 20%, 25%, and 30%, no collapse of the scaffold was observed with increasing strain, and the overall structures remained intact without failure, as shown in Figure 4a. In contrast, the P scaffolds with lower volume fractions of 10% and 15% were prone to structural collapse, leading to global failure [30]. Similarly, the IWP and G architectures at volume fractions of 20%, 25%, and 30% also exhibited no collapse and maintained structural integrity throughout deformation in Figure 4b,c. These results indicate that increasing the volume fraction effectively enhances the mechanical performance of the structures.

The stress–strain curves were obtained from experimental data of the tough resin scaffolds to investigate the influence of relative density on mechanical performance. For porous scaffolds with the same relative density, the pristine structure exhibits lower overall strength than the IWP scaffold. With increasing relative density, the peak strength of the P scaffold increases from 1.64 MPa to 4.32 MPa, while that of the G scaffold rises from 1.87 MPa to 4.53 MPa. The IWP scaffold shows the highest values, increasing from 2.58 MPa to 5.35 MPa, as shown in Figure 4d. These results clearly indicate a positive correlation between scaffold relative density and corresponding mechanical strength.

For the P structure with a volume fraction of 30%, the stress exhibits a sharp drop from a peak value of 4.63 MPa to 2.16 MPa in Figure 4a. This pronounced reduction contrasts strongly with the behavior observed at a volume fraction of 20%. It can be attributed to a transition in the failure mechanism from bending-dominated progressive collapse to a mixed tensile–bending brittle failure. In addition, the relatively thick struts lead to a more abrupt load redistribution once critical load bearing elements fracture, resulting in a rapid loss of load carrying capacity after the peak stress. In comparison, the P structure at 20% volume fraction is dominated by bending deformation with more distributed failure, resulting in a more gradual post-peak stress decline. At higher volume fractions, the G structure exhibits a more stable stress plateau compared with the P and IWP topologies, and the stress response at 30% volume fraction is noticeably smoother than that at 20% and 25%. The relatively gentle post-peak stress evolution of the G structure in Figure 4c suggests that, even for intrinsically brittle additively manufactured metamaterials, a denser G architecture can promote a more stable plateau response, thereby contributing to improved energy absorption capability in Figure 4e,f.

When comparing structures at the same volume fraction, the IWP topology consistently exhibits higher stress levels. After the initial stress drop, the IWP response develops a relatively stable plateau region in Figure 4b. In addition, this architecture demonstrates the highest energy absorption capacity across all tested densities. These observations clearly highlight the superior load-bearing and energy dissipation performance of the IWP design in Figure 4f. The specific results are shown in Table 2.

**Figure 5:**
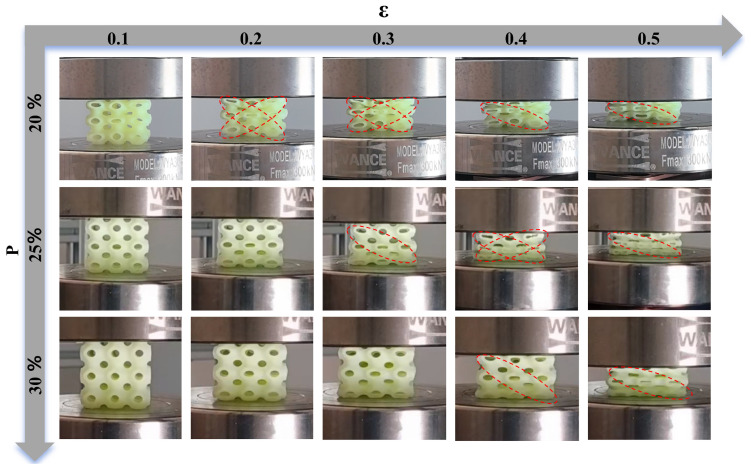
Overall deformation processes of P topology scaffolds with volume fractions of 20%, 25%, and 30%. The red dotted circles indicate localized failure regions.

**Figure 6:**
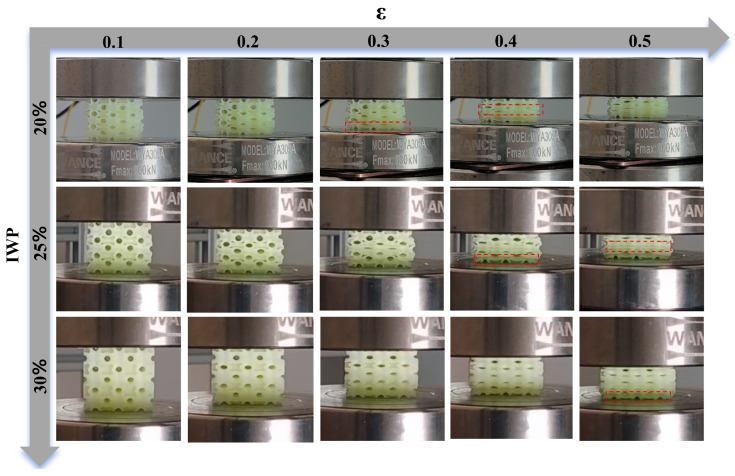
Overall deformation process of IWP topology scaffolds with volume fractions of 20%, 25%, and 30%. The red dotted circles indicate localized failure regions.

**Figure 7:**
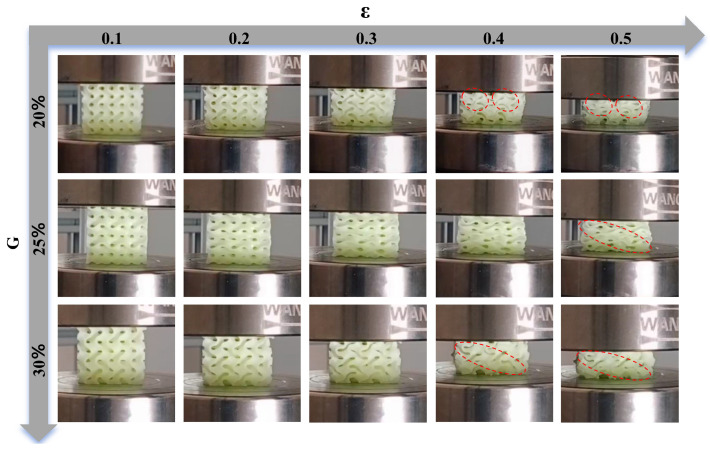
Overall deformation process of G topology scaffolds with volume fractions of 20%, 25%, and 30%. The red dotted circles indicate localized failure regions.

The deformation behaviors of the three single phase materials are shown in Figure 5, Figure 6 and Figure 7. Under compression, the P structure with a volume fraction of 20% develops a distinct 45° shear band at a strain of 0.1. However, at a higher volume fraction of 30%, no X-shaped shear band is observed at low strain levels in Figure 5.

In contrast, the IWP scaffolds with different relative densities exhibit a layer-by-layer progressive collapse failure mode in Figure 6. These results highlight the superior axial load bearing capacity and enhanced load distribution capability of the IWP scaffold compared with the original structures.

For the G structure, dispersed diagonal shear bands are formed at the lower volume fraction of 20%, which is associated with bending dominated deformation leading to distributed diagonal failure patterns. At the higher volume fraction of 30%, more localized deformation bands emerge, indicating a transition toward a combined tensile–shear deformation mode in Figure 7. The key advantage of the G architecture lies in its unique gyroid surface geometry, which promotes a more uniform stress distribution throughout the structure.

The investigation of monolithic materials reveals the intrinsic brittleness of additively manufactured metallic metamaterials, as well as their distinct structure-governed failure mechanisms. The following sections further compare the mechanical properties and deformation behaviors between the single phase materials and the interpenetrating phase composites (IPC).

### 3.3. Quasi-Static Mechanical Properties of IPC

**Figure 8:**
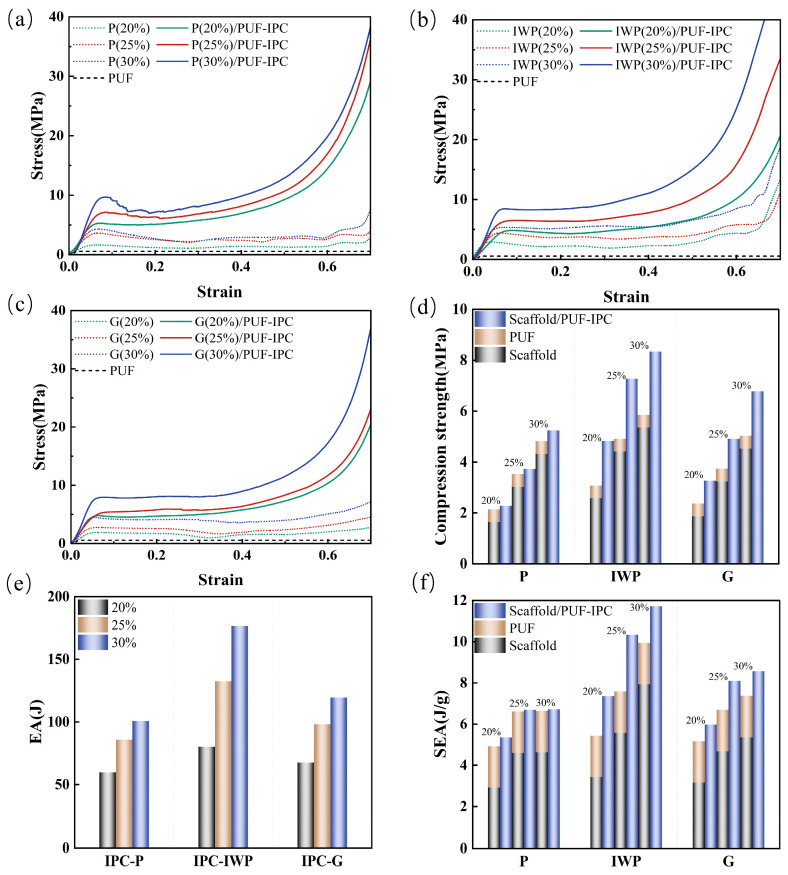
(**a**–**c**) Quasi-static compressive stress–strain curves of P, IWP, and G scaffolds, polyurethane matrix, and IPC materials. (**d**) Enhancement effect of IPC formation on peak strength. (**e**,**f**) Enhancement effect of IPC formation on energy absorption.

Figure 8 summarizes the compressive stress–strain responses, peak strength, and energy absorption characteristics of the tough resin, polyurethane matrix, and the corresponding interpenetrating phase composites (IPC). The volume fractions of the reinforcing phase are 20%, 25%, and 30%, consistent with the monolithic material tests shown in Figure 4. The stress–strain curves of the IPC exhibit pronounced differences compared with those of their individual constituent phases. Compared with the single-phase materials, the composites demonstrate significant improvements in both strength and toughness. This enhancement can be attributed to the interpenetrating architecture, which effectively suppresses rapid crack propagation.

As shown in Figure 8a–c, the stress–strain responses of the IPC can be divided into three distinct stages: the elastic region, the plastic plateau region, and the densification region. In the elastic stage, all IPCs exhibit a clear linear response at low strain levels, indicating coordinated deformation between the two phases. During the plastic plateau stage, the stress reaches a peak value and subsequently decreases. However, due to the constraint provided by the polymer matrix, the overall structural integrity is largely maintained. Notably, at a volume fraction of 30%, the stress level within the plateau region is occasionally lower than, or comparable to, that of IPC with lower reinforcement content for all three architectures. This phenomenon is most pronounced in the P-based IPC. At higher reinforcement fractions, the reduced volume of the confined polymer phase leads to a more gradual stress evolution during deformation.

For the P-based IPC with reinforcement volume fractions of 20%, 25%, and 30%, the peak stress reaches 1.64 MPa, 3.03 MPa, and 4.32 MPa, respectively, in Figure 8d. A similar positive correlation between reinforcement content and peak strength is also observed for the IWP and G-based IPC. Compared with the corresponding single phase materials at equivalent volume fractions, the IPC show strength improvements of 39.1%, 23.1%, and 21.3%, respectively, indicating that the strengthening effect gradually decreases with increasing reinforcement fraction. Although both the IWP and G architectures also exhibit a reduction in strengthening efficiency at higher volume fractions, the IWP-based IPC achieves peak strengths of 4.83 MPa, 7.27 MPa, and 8.34 MPa, corresponding to strength enhancements of 87.2%, 64.8%, and 55.9%, respectively, representing the highest overall improvement among the three configurations. The G-based IPC exhibits peak strengths of 3.27 MPa, 4.91 MPa, and 6.78 MPa, with corresponding increases of 74.9%, 51.5%, and 49.7%, respectively. It is noteworthy that IPC specimens with higher relative density of the tough resin phase consistently exhibit higher peak strength, indicating a positive correlation between the relative density of the resin scaffold and the load bearing capacity of the composite.

To clarify the synergistic effect of the two phases on energy absorption, Figure 8e,f compares the volumetric energy absorption of the monolithic scaffolds and the corresponding IPC across all architectures and volume fractions. Compared with the monolithic scaffolds, the IPC shows a significant enhancement in energy absorption. The IPC based on P, IWP, and G architectures all exhibit multiple fold increases at all three volume fractions, indicating a clear positive synergistic effect. For the IWP-based IPC, the specific energy absorption values at 20%, 25%, and 30% volume fractions are 7.359 J/g, 10.33 J/g, and 11.72 J/g, corresponding to increases of 114.54%, 85.51%, and 47.79%, respectively, in Figure 8f. For the G-based IPC, the values are 5.98 J/g, 8.01 J/g, and 8.57 J/g, with improvements of 89.24%, 72.99%, and 59.89%, respectively. For the P-based IPC, the specific energy absorption is 5.36 J/g, 6.69 J/g, and 6.71 J/g, corresponding to increases of 83.4%, 45.4%, and 45.01%, respectively. The superior performance of the IWP architecture can be attributed to the coupling between its inherent layer by layer collapse behavior and the energy dissipation of the foam phase, leading to a synergistic response greater than the sum of the individual phases. Overall, the energy absorption of the IPC increases with increasing reinforcement volume fraction, with the 30% IWP IPC achieving the highest value of 11.72 J/g. The specific results are shown in Table 3.

**Figure 9:**
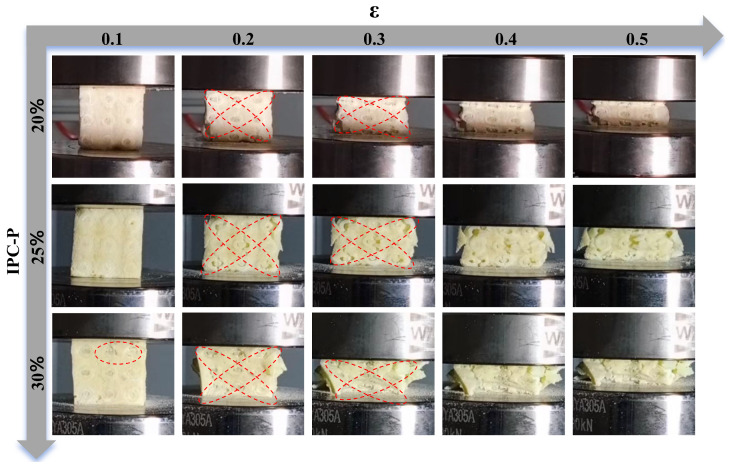
Overall deformation process of IPC-P topology scaffolds with volume fractions of 20%, 25%, and 30%. The red dotted circles indicate localized failure regions.

For the P-IPC structures, the specimen with 30% volume fraction exhibits faint diagonal microcracks at a strain of 0.1. In contrast, no visible surface cracking is observed for the other volume fractions, as well as for all IWP-based IPC at the same strain level. When the strain reaches 0.2, all P-based IPC develop distinct X-shaped shear bands. With increasing reinforcement content, these shear bands become more pronounced, accompanied by more severe crack propagation in Figure 9.

**Figure 10:**
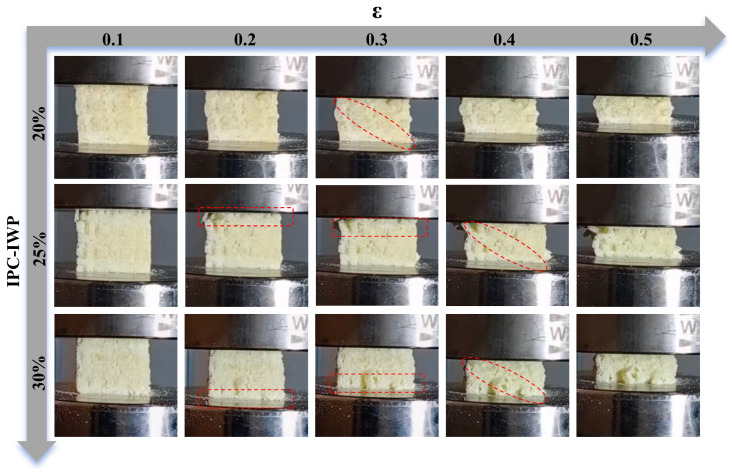
Overall deformation process of IPC-IWP topology scaffolds with volume fractions of 20%, 25%, and 30%. The red dotted circles indicate localized failure regions.

In the IWP-IPC, the specimen with 20% volume fraction shows no obvious surface cracking. In contrast, both the 25% and 30% specimens exhibit clear transverse fracture features. As the strain increases from 0.3 to 0.5, cracks in the P-based IPC propagate along the X-shaped shear bands, and the 30% specimen shows the earliest through-thickness fracture. For the IWP-based IPC, diagonal shear failure appears at a strain of 0.3 in the 20% specimen; however, due to its relatively thin wall geometry, the damage is less evident. The 25% and 30% specimens exhibit more pronounced diagonal failure characteristics. Beyond a strain of 0.5, all stress–strain curves enter the densification stage in Figure 10.

**Figure 11:**
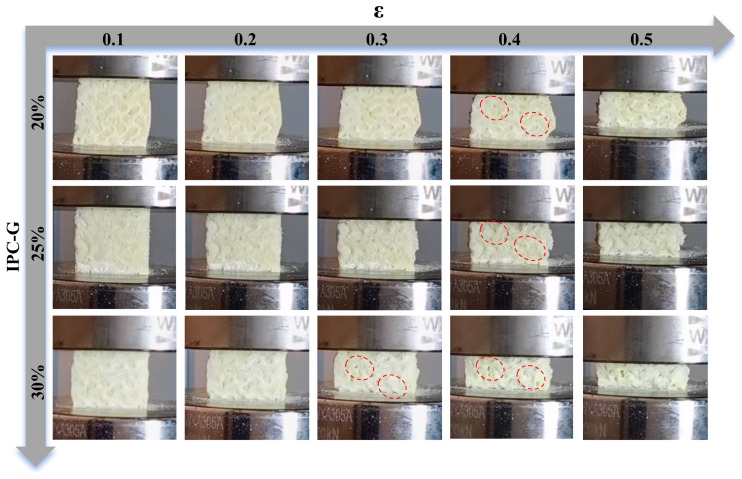
Overall deformation process of IPC-G topology scaffolds with volume fractions of 20%, 25%, and 30%. The red dotted circles indicate localized failure regions.

For the G-IPC, crack distribution is relatively uniform across all three volume fractions, which can be attributed to the inherently smooth curvature of the gyroid structure that promotes more uniform load diffusion and reduces stress concentration. The 30% G-based IPC shows the earliest onset of distributed cracking at a strain of 0.3. In general, lower volume fractions correspond to a higher content of the PUF phase, resulting in less pronounced cracking at the same strain level in Figure 11.

## 4. Conclusions

This study systematically investigated the effects of three representative TPMS topologies, namely P, IWP, and G, together with three reinforcement volume fractions (20%, 25%, and 30%), on the mechanical properties and deformation behavior of monolithic lattice structures and their corresponding interpenetrating phase composites (IPC) under quasi-static compression.

(1) Both topology and volume fraction have a pronounced influence on the compressive performance of the monolithic lattice structures. The IWP architecture exhibits the most favorable overall mechanical response. The P topology is prone to shear-induced failure at low volume fractions, while the G structure shows a more uniform and integrated deformation and fracture mode. Increasing the volume fraction generally enhances the strength and plateau stability of all configurations.
(2) The IPC design effectively suppresses brittle fracture and transforms shear instability into a progressive collapse mode, thereby significantly improving yield strength and energy absorption capacity.
(3) Comparison with the linear superposition results indicates that both peak stress and plateau toughness of IPC are higher than the theoretical sum of the resin scaffold and PUF phases, confirming a clear synergistic strengthening effect. The tough resin scaffold provides the primary load-bearing framework, while the PUF phase offers local support and energy dissipation within the porous network. The interaction between the two phases enables improved load stability and energy absorption while maintaining lightweight characteristics.
(4) At lower reinforcement volume fractions, the contribution of the matrix phase to mechanical enhancement is more pronounced, providing practical guidance for the design of lightweight and energy absorbing porous IPC materials.

## Figures and Tables

**Table 1:** Measured density and relative density of the resin scaffolds and corresponding IPC specimens.

Topology	Designed Volume Fraction (%)	Scaffold Density (g/cm^3^)	Relative Density (%)	IPC Density (g/cm^3^)
P	20	0.228	19.8	0.348
P	25	0.284	24.7	0.397
P	30	0.341	29.7	0.446
IWP	20	0.232	20.2	0.352
IWP	25	0.289	25.1	0.401
IWP	30	0.347	30.2	0.451
G	20	0.235	20.4	0.355
G	25	0.292	25.4	0.405
G	30	0.351	30.5	0.455

**Table 2:** Mechanical properties of TPMS scaffolds under quasi-static compression.

Topology	Designed Volume Fraction (%)	Compression Strength (MPa)	EA (J)	SEA (J/g)
P	20	1.64	18.27	2.92
P	25	3.03	36.70	4.60
P	30	4.32	44.36	4.63
IWP	20	2.58	21.13	3.43
IWP	25	4.41	44.29	5.57
IWP	30	5.35	77.27	7.93
G	20	1.87	19.54	3.16
G	25	3.24	36.99	4.68
G	30	4.53	51.42	5.36

**Table 3:** Mechanical properties of IPC under quasi-static compression.

Topology	Designed Volume Fraction (%)	Compression Strength (MPa)	EA (J)	SEA (J/g)
IPC-P	20	2.28	59.83	5.36
IPC-P	25	3.43	85.81	6.68
IPC-P	30	5.24	100.71	6.71
IPC-IWP	20	4.83	80.25	7.36
IPC-IWP	25	7.27	132.47	10.33
IPC-IWP	30	8.34	176.57	11.72
IPC-G	20	3.27	67.62	5.98
IPC-G	25	4.91	98.18	8.09
IPC-G	30	6.78	119.45	8.57

## Data Availability

The original contributions presented in this study are included in the article. Further inquiries can be directed to the corresponding author.
